# Reproductive and Fertility Care of Women with Schizophrenia: A Narrative Review

**DOI:** 10.3390/healthcare13182349

**Published:** 2025-09-18

**Authors:** Alexandre González-Rodríguez, José Antonio Monreal, Mentxu Natividad, Jesús Cobo, Bruma Palacios-Hernández, Leah C. Susser

**Affiliations:** 1 Department of Mental Health, Mutua Terrassa University Hospital, Fundació Docència I Recerca Mutua Terrassa, University of Barcelona (UB), 5 Doctor Robert Square, 08221 Terrassa, Spain; mnatividad@mutuaterrassa.cat; 2 Centro de Investigación Biomédica en Red de Salud Mental (CIBERSAM), Instituto de Salud Carlos III, 3-5 Calle Monforte de Lemos, Pabellón 11, Planta 0, 28029 Madrid, Spain; 3 Institut de Neurociències, Universitat Autònoma de Barcelona (UAB), 08221 Terrassa, Spain; 4 Department of Mental Health, Parc Taulí University Hospital, Autonomous University of Barcelona (UAB), 1 Parc Taulí, 08208 Sabadell, Spain; jcobo@tauli.cat; 5 Perinatal Mental Health Research Laboratory CITPsi-UAEM, Autonomous University of the State of Morelos, 1001 Universidad Avenue, Colonia Chamilpa, Cuernavaca 62350, Mexico; bruma.palacios@uaem.mx; 6 Weill Cornell Medicine, 21 Bloomingdale Road, White Plains, NY 10605, USA; lcs7001@med.cornell.edu

**Keywords:** schizophrenia, psychosis, pregnancy, postpartum, contraception, family planning, fertility

## Abstract

Women with schizophrenia have specific reproductive and fertility care needs that differ from those of men. This narrative review aims to explore the reproductive and fertility care needs of women with schizophrenia related to fertility, menstrual cycle, family planning, and the perinatal period. Hormonal fluctuations in the menstrual cycle can significantly affect clinical symptoms and the efficacy of contraceptive treatments in women with schizophrenia. When undergoing fertility treatment, attention should be given to the potential interactions between fertility treatment drugs and antipsychotics, as well as the potential impact of antipsychotics on the ability to conceive. Although pregnancy rates and participation in fertility programs have increased among women with schizophrenia, very little information is available on the safety and efficacy of fertility treatments in women with severe psychotic disorders. This is an area that warrants further study. Mobilizing social supports and optimizing medication are important components of the treatment of schizophrenia in the perinatal period and are important for the health and safety of infants and mothers. In conclusion, preventing relapse and promoting physical and mental health in this group of women during successive reproductive periods of life is vital and requires an emphasis on the social and psychiatric context, as well as the biological changes occurring at these times.

## 1. Introduction

Schizophrenia is a serious mental disorder with a high burden of disease in men and women and similar prevalence between the sexes [1,2]. However, epidemiology, symptomatology, and comorbidity differ between the sexes. These sex-specific differences must be recognized and incorporated into treatment to decrease disparities in healthcare for women with schizophrenia.

The course of schizophrenia in men and women is different. Men diagnosed with schizophrenia show an earlier age of disorder onset than women, while women present a second peak of incidence at the time of menopause [3]. Consequently, psychiatric intervention needs to be tailored to the sex-specific biological circumstances of their life stages.

Characteristic symptoms in men and women with schizophrenia dictate the appropriate interventions. Men with schizophrenia suffer more severe negative and cognitive symptoms than women, which may be associated with hormone levels and psychosocial risk factors [1]. Women, on the other hand, generally present with more frequent hallucinations, delusions, and depressive symptoms [4]. During reproductive years, clinical outcomes favor women, who show a higher response to antipsychotic medications [3]. Compared to men, women demonstrate better social functioning, partly due to neuroprotection from estrogen during their reproductive years and a higher ability to mitigate symptoms of schizophrenia [1,2,3,4]. In brief, the estrogen protection hypothesis of schizophrenia postulates that estrogens are neuroprotective and interact with the main neurotransmitter pathways. This results in a later development of schizophrenia in women and experiencing less severe psychotic symptoms than men [2,4].

The prognosis in women, however, changes after menopause. At the time of menopause, psychosis in women becomes more treatment-refractory, the requirement for antipsychotic doses is higher, and adverse events are more common [5]. The risk of tardive dyskinesia in women, for example, increases at this time [6]. Neuroprotection conferred by estrogen, which drops considerably at menopause, is responsible for these changes [7]. The impact of estrogen on schizophrenia course in women highlights the unique care needs of women with schizophrenia across their lifespan and the importance of taking into consideration the reproductive phase in treatment [8,9].

Although both men and women with schizophrenia show social risk factors that affect the prognosis of the disorder, these factors impact women more than men [10,11]. These factors include isolation, social exclusion, discrimination, immigration, living in an urban area, poverty, trauma, inadequate housing, and human trafficking [10,11].

Women with schizophrenia have sex-specific gynecologic and obstetric care needs that remain inadequately addressed in current medical systems. They are less likely to receive medical screening, including Human Immunodeficiency Virus (HIV) testing, regular gynecologic examination, cervical cancer screening, and family planning, than females without schizophrenia [9]. Furthermore, many women with schizophrenia are not asked about their sexual health [9]. Unique reproductive and fertility health concerns in women with schizophrenia, including those related to pregnancy, menstruation, and fertility, need to be recognized and addressed. Approximately 50% of women with schizophrenia become mothers and care for children; consequently, it is imperative for clinical settings to target reproductive health [2]. Unfortunately, reproductive health in women with schizophrenia is an understudied area, and very few studies have focused on the reproductive and fertility needs of women with schizophrenia.

### Aims

The goal of this narrative review is to explore the reproductive and fertility care needs of women with schizophrenia. Our aim was to address the following questions:

(1) How do menstrual cycle phases impact clinical course in women with schizophrenia?
(2) What are the important variables to recognize related to contraception and family planning in women with schizophrenia?
(3) How effective and safe are fertility treatments for women with schizophrenia?
(4) What are the clinical needs of women with schizophrenia during pregnancy and the postpartum period?

## 2. Methods

### 2.1. Screening and Selection of Evidence in Women with Schizophrenia

We carried out a narrative review focused on reproductive and fertility care needs in women with schizophrenia. The PubMed database was searched with the following search terms: Schizophrenia AND (“menstrual cycle” OR “family planning” OR “contraception” OR “fertility treatments” OR “maternal health needs”). The menstrual cycle, family planning, and contraception were reviewed in order to introduce the topic of fertility treatments. Finally, maternal health needs were reviewed, specifically for women with schizophrenia suffering from infertility. We included papers published within the last ten years. Classic papers in the field were also included if they were considered a reference point in the field.

We conducted a narrative review because it provides a flexible approach that allowed us to synthesize and give an overview of the limited available literature across this broad topic and to identify research gaps. Narrative reviews are helpful for exploring complex subjects such as reproductive and fertility care and family planning in women with schizophrenia and for generating hypotheses for future studies and clinical programs.

Our main goal was to integrate the fragmented literature in this field, generate hypotheses for future research and clinical programs, and provide a general overview of the topic for learning purposes.

### 2.2. Inclusion and Exclusion Criteria

Papers were included if they met the following criteria: (1) study participants were women suffering from schizophrenia or closely related disorders, (2) there was clinical information on menstrual cycle, family planning, fertility treatments, perinatal, or parenting needs, and (3) the language was English, French, Spanish, or German. Cohort studies, clinical trials, and other forms of research studies, as well as reviews, were included if judged by the authors to be of good methodologic quality. Case reports were excluded.

### 2.3. Reporting of Results

We grouped the included papers into five categories: (1) menstrual cycle in women with schizophrenia, (2) family planning and contraception considerations, (3) fertility in women with schizophrenia, and (4) perinatal period in women with schizophrenia.

## 3. Results

Assessment of menstrual cycle abnormalities and menstrual cycle-related symptom patterns is crucial when evaluating women with schizophrenia. In women of reproductive age, discussions about family planning are vital and should be followed up with discussions regarding contraception and/or conception, depending on a woman’s family planning preferences. In women who are planning to conceive, infertility should be carefully addressed, and, when relevant, fertility treatment options should be discussed. Furthermore, discussion of options for schizophrenia treatment in pregnancy and postpartum is imperative for mitigating risks to the mother and child. This discussion should include the important roles of antipsychotic medication, support systems, and protection of sleep in the perinatal period.

### 3.1. Menstrual Cycle in Women with Schizophrenia

Women with schizophrenia experience fluctuations in symptoms across the phases of their menstrual cycle. This is due to the effect of hormones, both directly on psychotic symptoms and also on antipsychotic blood levels.

Certain, but not all, women with schizophrenia can experience fluctuations in psychotic symptom severity with changes in estrogen levels. During phases of the menstrual cycle when estrogen levels are low, psychotic symptom severity can increase; when estrogen in the bloodstream is elevated during the mid-luteal phase, symptom severity can decrease [4,12,13]. In addition, certain women can experience menstrual psychosis, in which they only experience psychotic symptoms during menstruation, when estrogen levels are low [14].

Estrogens are known to have neurotrophic and neuroprotective effects on the brain [8]. This also explains why women are more vulnerable to developing non-affective psychosis and worsening of pre-existing psychotic symptoms when estrogen levels decline after menopause [15,16].

Gonadal steroid levels can also alter enzyme activity involved in the metabolism of antipsychotic medications, thereby increasing or decreasing blood levels of certain antipsychotic drugs. For example, estrogen increases the levels of olanzapine and clozapine by inhibiting the metabolism of these drugs by the enzyme Cytochrome P450 1A2 (CYP1A2); it decreases the levels of quetiapine through its effects on the enzyme Cytochrome P450 3A4 (CYP3A4) [16]. These changes lead to alterations in antipsychotic plasma levels across the menstrual cycle.

Conversely, antipsychotic treatment may also affect hormone levels by increasing prolactin levels. The risk of hyperprolactinemia varies between antipsychotic drugs [17]. Hyperprolactinemia leads to galactorrhea and lowers estrogen levels, resulting in amenorrhea or oligomenorrhea, and infertility [18,19,20]. Understanding the relationship between the menstrual cycle and antipsychotic medications can help mitigate the risk of relapse.

### 3.2. Contraception and Family Planning in Women with Schizophrenia

Historically, women with schizophrenia have had lower fertility rates than other women, possibly due to hyperprolactinemia caused by first-generation antipsychotic medications and the impact of negative symptoms on relationships [21]. However, birth rates in women with schizophrenia are increasing, probably due to both the use of antipsychotics that have less effect on prolactin and the effectiveness of current treatments for schizophrenia [21]. Although pregnancy rates in women with schizophrenia have increased, many women with schizophrenia continue to struggle with fertility problems, and recognition of risk factors allows for targeted fertility treatment and family planning assistance.

The discussion of family planning and sexuality is important for all reproductive-age women, including women with schizophrenia [22]. These discussions should include family planning preferences, contraceptive options if the woman does not want to conceive at this time, and contraceptive efficacy [23]. Furthermore, this discussion should include coagulation risk due to the antipsychotic-induced metabolic side effects and smoking prevalence in schizophrenia. The potential impact of exogenous estradiol on antipsychotic levels is another relevant factor to be considered in women. Estradiol-containing oral contraceptives may increase clozapine levels and risk of toxicity [24,25,26]. Antipsychotic dose may need to be adjusted when an oral contraceptive is added or discontinued.

Contraceptive efficacy is an important consideration. Women with schizophrenia are at high risk of unplanned pregnancy and rapid repeat pregnancy [27,28]. In addition, women experiencing their first episode of psychosis have high rates of inconsistent contraceptive adherence [29,30]. Therefore, long-acting reversible contraceptives and contraceptive injections may be appropriate options to help with adherence.

For women with schizophrenia who wish to become pregnant, healthcare providers should engage in discussion about relationships, parenting, and treatment options and preferences for a future perinatal period. This conversation should include discussion of the impact of antipsychotics on fertility, efficacy of alternative antipsychotic regimens that do not raise prolactin levels for the individual, relapse prevention, and mobilization of family support in the perinatal period [20].

Due to the high rates of unplanned pregnancy, a discussion of treatment options in pregnancy should be offered to all women of reproductive age with schizophrenia [29,30]. Medication is often a necessary part of the treatment of schizophrenia. As a result, the discussion should include information about antipsychotics in pregnancy and breastfeeding and the risks of relapse to the mother and fetus. This discussion may reduce the risk of abruptly stopping medication during pregnancy, which would increase the risk of relapse and impact the well-being of the expectant mother and her fetus.

While certain antipsychotics have been well studied with overall reassuring findings in pregnancy and are recognized to be generally low risk (haloperidol, olanzapine, quetiapine, risperidone, and aripiprazole) [31,32], others have not been well studied. If a woman with schizophrenia who responded best to a medication less studied in pregnancy (e.g., clozapine, a long-acting injectable formulation, or a new antipsychotic) becomes pregnant, the unknown risks should be weighed against the benefits of the medication for her severe mental disorder. In many cases, continuing the antipsychotic to which she responded is important to prevent the consequences of worsening psychosis in the perinatal period. The risks of not treating her disorder for maternal and fetal health in pregnancy should not be underestimated.

Interventions to increase support networks can help mitigate the risk of worsening the disorder and help her transition to motherhood. Each woman’s needs must be assessed individually. Mobilization of social supports is essential, including linking her to available resources for pregnant and new mothers, involving family members, and helping the expectant mother to take prenatal vitamins, stop smoking, attend prenatal appointments, create a plan to protect sleep postpartum, and with childcare. Close monitoring of symptoms through pregnancy and the postpartum period is important. Sleep disorders should be evaluated, and early interventions should be offered.

The postpartum period is a particularly high-risk period for psychosis relapse due to the drastic drop in estrogen after delivery, sleep deprivation, and the new responsibility of caring for an infant [33].

Table 1 summarizes the main topics of concern about family planning in schizophrenia.

### 3.3. Fertility Treatments in Women with Schizophrenia

Simoila and collaborators [34] investigated pregnancy, delivery, and postpartum-related outcomes of women with schizophrenia and schizoaffective disorder compared to women in the general population. They found that women with schizophrenia have fewer pregnancies than women in the general population.

High levels of stress, the impact of illness on relationships, antipsychotic side effects (i.e., Hyperprolactinemia), and medical and psychiatric comorbidity can impact fertility in women with schizophrenia [20,35,36]. Unfortunately, disparities in access to fertility care exist. Access to fertility treatment is low in this population for economic and stigma-related reasons [37,38].

#### 3.3.1. Use of Assisted Reproductive Technology (ART) Programs

Few women with schizophrenia participate in ART programs (although exact numbers are unknown), and very few studies have specifically investigated the safety and efficacy of fertility treatments in women with schizophrenia.

Yli-Kuha et al. studied a cohort of 9175 women who had undergone in vitro fertilization (IVF), intracytoplasmic sperm injection (ICSI), or frozen embryo transfer (FET) in Finland [39]. They used a control group (n = 9175) matched for age and municipality and further adjusted for marital status and socioeconomic status [39]. Very few women with schizophrenia were included in the study [39]. Hospitalizations for psychotic disorders were higher among controls compared to the group receiving fertility treatment, which suggests lower access to ART in women with a psychotic disorder.

In a cohort of 42,915 Danish women receiving assisted reproductive treatment (ART) between 1994 and 2009, ref. [40], only 244 women were diagnosed with a psychotic disorder before, during, or after ART treatment. The authors emphasized that the prevalence of women with psychotic disorders undergoing fertility treatment is low compared to the general population.

#### 3.3.2. Safety of Fertility Treatments

Few studies have examined the safety of fertility treatments specifically in women with schizophrenia. Women with schizophrenia are often excluded from studies of fertility treatments, limiting the generalizability of both efficacy and safety to this population. Areas for study include the potential interactions between medications used in ART and antipsychotic medications, and also whether ART is associated with the risk of relapse of schizophrenia.

Medications used in fertility treatment and antipsychotic drugs can have pharmacodynamic and pharmacokinetic interactions [41]. Cytochrome P450 is responsible for the metabolism of antipsychotic medications, and its activity impacts the plasma levels of antipsychotics and their metabolites [42,43]. Cytochrome P450 2D6 (CYP2D6), Cytochrome P450 1A2 (CYP1A2), and Cytochrome P450 3A4 (CYP3A4) are the main enzymes implicated in the metabolism of antipsychotic drugs [44]. Medications that affect the activity of these cytochrome P450 enzymes could alter antipsychotic plasma levels. Conversely, antipsychotic medications can change the activity of cytochrome P450 enzymes as well. Certain antipsychotic medications are inhibitors of CYP2D6 [45,46]. Clomiphene, a treatment to promote ovulation, is metabolized by CYP2D6. Antipsychotic medications that are CYP2D6 inhibitors could decrease the metabolism of clomiphene and result in higher concentrations of clomiphene, which could impact efficacy and risk of adverse events [47].

In a cohort of 98,320 women referred for treatment, Baldur-Felskov and collaborators [48] examined the impact of fertility treatment outcomes on the risk of mental disorders, including schizophrenia and related psychotic disorders. Women not giving birth after the infertility treatment compared to those who gave birth showed a higher risk of hospitalization for all psychiatric disorders, alcohol abuse, and schizophrenia and other psychotic disorders [48].

#### 3.3.3. Effectiveness of Fertility Treatments

Psychopharmacological treatment of schizophrenia (particularly antipsychotics) may be relevant to the effectiveness of ART programs. Certain antipsychotics can induce hyperprolactinemia, which impacts fertility. Hyperprolactinemia may need to be reversed by switching to prolactin-sparing antipsychotics prior to ART [49].

Table 2 summarizes the main findings regarding fertility treatments in women with schizophrenia.

### 3.4. Pregnancy and Postpartum Health Needs in Women with Schizophrenia

Women diagnosed with schizophrenia have reported that being a mother is the most important thing in their lives, as it provides meaning and focus, and children motivate them to stay healthy [50]. At the same time, motherhood is a heavy responsibility when added to the needs of their psychiatric disorder and associated socioeconomic risk factors [51].

In a study of 75 women with psychotic disorders in England, the authors compared women with psychosis who had children with those who did not [52]. The authors found that having a child was associated with a later age of onset of the disorder and higher rates of housing problems, suggesting that women with psychosis who have children have different needs than those who do not.

#### 3.4.1. Prevention and Treatment of Psychotic Disorder Relapse in Postpartum Women with Schizophrenia

For mothers with schizophrenia who have young children, close monitoring can mitigate the risk of relapse and associated consequences. Maintenance of antipsychotic and psychological treatment and monitoring of parental stress require frequent clinical assessment [53]. Early signs of relapse, such as insomnia, poor hygiene, and increased suspiciousness, require intervention [54].

#### 3.4.2. Special Attention in the Perinatal Period

The postpartum period is a particularly vulnerable time for women with schizophrenia. This period is associated with increased risk of relapse and hospitalization [55].

Seeman reports that mothers with schizophrenia and other psychotic disorders may present with perinatal syndromes such as delusional denial of pregnancy, postpartum psychosis, and postpartum delusional misidentification syndrome [56]. It is important to emphasize that a severe psychotic episode during the postpartum period is considered a psychiatric emergency requiring urgent attention and treatment and is associated with filicide and maternal suicide [57]. Postpartum psychosis can be a part of bipolar disorder, or postpartum psychosis can be a relapse of a chronic psychotic disorder such as schizophrenia. The recommended intervention is the hospitalization of the mother in a psychiatric unit with her baby, if possible. Mother–baby units are the most specialized intervention to improve the mental health of mothers with psychotic episodes in the perinatal period, where a well-trained multidisciplinary team can provide 24 h attention to the mother and help her with the care of her baby. This can include family visits, psychoeducation, and training in parenting skills. Unfortunately, mother and baby units are still scarce in many countries, particularly low- and middle-income countries. A systematic review analyzed the results of the effectiveness of mother-baby units in different countries. Benefits were reported for women with schizophrenia, although their recovery was poor compared to mothers with other diagnoses [58].

A recent study reported that psychological interventions for women with postpartum psychosis can help promote trust, improve feelings of connectedness, re-establish relationships, process feelings of loss, manage fear of relapse, and plan for their future [59]. While the prognosis after an episode of isolated postpartum psychosis is good, to date, current studies have reported that women with a long history of psychotic disorders, such as schizophrenia, recover less well from postpartum psychosis than do other women [50].

#### 3.4.3. Support for the Mother-Infant Relationship

Women with schizophrenia require additional support and parental training in practical baby care skills. They may at first be insensitive to their infant’s needs and need encouragement to bond with their infants [56,60].

The fear of custody loss is one of the most frequent concerns reported by women with schizophrenia caring for children [50,51,56]. Fear of losing their children can prevent open communication with healthcare professionals [50].

Parenting assessment and support services need to be a public health priority. The antipsychotic side effect of blunted affect can be mistaken for a lack of interest in their child [51,60].

#### 3.4.4. Reduction in Stigma

Women with schizophrenia who become mothers face stigma. Many members of society believe that their disorder deprives them of the ability to be good enough mothers or to care for their children [50,51]. To reduce the stigma while attending to the needs of mothers with schizophrenia, we recommend several actions. These actions include the implementation of mental health campaigns and mental health training for primary care professionals and social services teams. A less judgmental and more empathetic approach by health and social services staff can help these mothers more openly discuss their needs and help address their challenges, such as social isolation.

#### 3.4.5. Economic, Occupational, and Vocational Help

Linking mothers with schizophrenia to services that provide income supplementation (e.g., loans, food banks, safe housing options) can help them to meet basic living needs [56]. Alleviating financial stress helps mothers with schizophrenia concentrate on caring for their children and can also reduce stress, which reduces the risk of exacerbation of psychiatric symptoms. Table 3 presents the main findings of the studies focusing on maternal health needs in women with schizophrenia.

**Figure 1:**
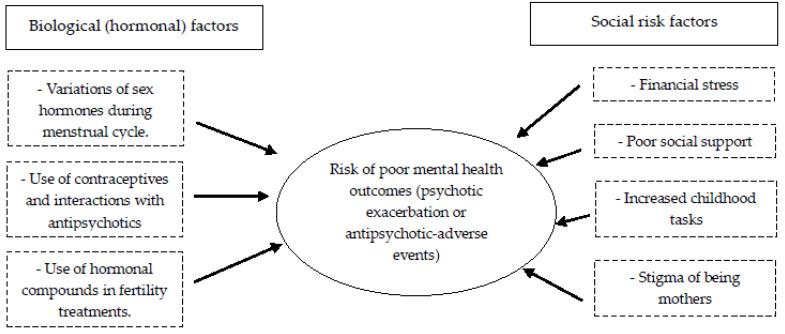
Risk factors for worsening clinical course in women with schizophrenia that should be considered during family planning discussions.

Figure 1 illustrates biological and social risk factors for the worsening clinical course of women with schizophrenia.

## 4. Discussion

Women with schizophrenia have specific reproductive and fertility care needs that differ from those of men and that need to be considered when providing healthcare [61].

Before menopause, certain women with schizophrenia experience symptom fluctuation across the menstrual cycle, which has been attributed to the effects of hormones both directly on psychotic symptoms and on the metabolism of antipsychotic medication [4,12]. Recent studies have highlighted the effects of menstrual cycle phase on psychotic symptom course [62]. For example, psychiatric hospitalizations in women with schizophrenia are more frequent when estrogen levels are low in the late premenstrual/early menstrual phase of the cycle [14]. This neuroprotective effect of estrogens in schizophrenia is observed throughout a woman’s life and is consistent with the estrogen-protection hypothesis of schizophrenia, which has been supported by many authors [4].

Family planning and contraception are important areas to discuss with women with schizophrenia. Improving access to family planning services for women with schizophrenia is crucial. This enables women to make informed decisions about reproduction, including contraceptive choice and family planning. A recent cross-sectional study in women suffering from mental disorders found that injectable and oral contraceptive pills were the most commonly used methods of contraception in those women [63]. Half of the women participating in the study used at least one family planning method at the time of the study inclusion. Coordination of care between mental health and gynecological services when discussing family planning with women with schizophrenia is recommended [64]. This allows discussion of interactions between contraceptive methods and antipsychotic medications for women who would like to prevent conception and discussion of appropriate treatment plans and monitoring for women who would like to conceive. Estrogen in oral contraceptives impacts antipsychotic metabolism, and antipsychotic dosing may need to be adjusted when starting or discontinuing antipsychotic medications [65].

When treating women with schizophrenia who would like to conceive, common causes of infertility in this population of women should be evaluated. One common cause of infertility in women with schizophrenia is hyperprolactinemia, which is often associated with antipsychotic medication use [17,20]. However, the selection of an antipsychotic that does not raise prolactin levels can prevent this cause of infertility. For example, aripiprazole has been found to effectively and safely reduce prolactin levels in women with schizophrenia [66]. Polycystic ovary syndrome (PCOS) is another common cause of infertility in women with schizophrenia. A recent study investigated the shared molecular mechanisms underlying the etiology of schizophrenia and polycystic ovary syndrome [67]. The authors identified common biomarkers for both disorders.

Birth rates are increasing in women with schizophrenia, in part due to the use of antipsychotics that affect prolactin less and in part due to improved treatment of schizophrenia [21]. Recent studies report a frequency of maternity in women with schizophrenia of around 50% [68]. As a result, discussions about family planning with reproductive-aged women with schizophrenia are imperative both to help achieve their family planning goals and preferences and to help maintain stability in the perinatal period.

The use of fertility treatments is low among women with schizophrenia. Furthermore, there is a lack of data regarding the safety and effectiveness of such treatments for these women. Potential drug interactions should be considered when combining fertility drugs and antipsychotics. Around 30–50% of antipsychotics are metabolized by CYP2D6, which is also the main enzyme responsible for metabolizing clomiphene, a treatment to promote ovulation [47]. Dose adjustment may be required [16,17,18,19,20,21,22,23,24,25,26,27,28,29,30,31,32,33,34,35,36,37,38,39,40,41,42,43,44].

Women with schizophrenia are frequently excluded from studies on fertility treatments due to concerns about the potential impact of fertility medications on mental health symptoms and because of the lack of data on the safety of these treatments in this population of women with severe mental disorders [49]. The limited access to fertility treatment programs for women with schizophrenia is likely due to social risk factors [49]. The document of the European Society of Human Reproduction and Embryology (ESHRE) for the Preimplantation Genetic Testing (PGT) Consortium Steering Committee [69] details inclusion and exclusion criteria before PGT. In the document, it is stated that PGT should be carefully considered if one of the partners suffers from severe physical or psychological problems. Due to exclusion from studies, the generalizability of studies on ART to women with schizophrenia and other mental disorders is unknown.

Likely barriers for women with schizophrenia to access fertility treatment programs include socioeconomic factors (e.g., socioeconomic status, insurance, and education level), language, racial disparities, and culture [70,71,72]. Comorbid medical conditions, including those related to lifestyle factors associated with schizophrenia, may also limit the ability to participate in fertility treatment programs. Other possible barriers to accessing fertility treatment for women with schizophrenia may include exclusion criteria for protocols and stigma.

Women with schizophrenia from low-income countries may experience further barriers to accessing evidence-based fertility treatment. In low-income countries, access to infertility treatments is often limited or unavailable [73]. In low-income countries, advanced diagnostic and treatment services are mostly provided by the private sector, to which women suffering from severe mental disorders with other social risk factors often do not have access. Furthermore, in certain cultures, the use of traditional and religious healers to address infertility is more common, and access to medical fertility treatments is low [73]. The implementation of low-cost and accessible fertility clinics in low- and middle-income countries should be a priority to improve access to evidence-based fertility care and to reduce disparities in care.

Close collaboration between reproductive health interventions and mental health providers, including co-located services, may improve access to fertility treatments for women with schizophrenia with infertility who would like to conceive and become mothers, and it can also improve mental health outcomes in the perinatal period [74,75].

Women with schizophrenia caring for young children have specific healthcare needs that require multidisciplinary approaches. The postpartum period is a period of increased vulnerability for women with schizophrenia. In addition to psychopharmacologic treatment, mobilization of social supports, parenting supports, healthy diet, sleep hygiene, and exercise can help reduce the risk of psychotic exacerbation in women with schizophrenia in the perinatal period and operate as preventive interventions [54,76].

In summary, women with schizophrenia have specific reproductive and fertility care needs compared to men. These specific health needs should be assessed and addressed in specialized healthcare settings where possible [77].

### Limitations and Strengths

This review has several limitations. The majority of studies of schizophrenia course across reproductive events did not assess hormone levels, which would be important for evaluating the potential associations between hormonal changes and clinical symptoms. Furthermore, few studies examined the use of fertility treatments in women with schizophrenia. This limits the generalizability of the efficacy of fertility programs to women on antipsychotic medications and the safety of fertility treatments to women with schizophrenia. Currently, limited literature exists on the reproductive and fertility care needs of women with schizophrenia, and this review highlights the need for future studies in this area to support women with schizophrenia as they make decisions regarding family planning and contraception and to optimize ways to maintain stability through the perinatal period.

## 5. Conclusions

This review highlights reproductive and fertility care needs that require attention for women with schizophrenia. Changes in hormone levels across the menstrual cycle can impact clinical symptoms in women with schizophrenia. Sex hormones have a direct effect on psychotic symptoms, and they also affect blood levels of some, but not all, antipsychotic medications. Certain hormonal contraceptive medications can impact antipsychotic medication levels as well. For women with schizophrenia who would like to conceive, recognition of the impact that certain antipsychotic medications can have on the ability to conceive is imperative. There is little information about the safety and overall effectiveness of fertility treatments in women with schizophrenia, and this is an area that warrants study. Finally, continued psychiatric treatment and establishing support for the mother during the perinatal period help reduce the risks of a worsening clinical course and are important for the health of the mother and the child. Close collaboration between the obstetric and mental health teams can improve monitoring and medical care during the perinatal period.

Future studies should focus on identifying and reducing barriers that women with schizophrenia face when accessing infertility treatment. Future studies should also investigate the safety and efficacy of fertility treatments in this population. Findings from these studies can then inform policies to reduce disparities in access to fertility care. Programs to help reduce stress, increase socialization, and provide educational support for parenting are important components of treatment during the perinatal period.

## Figures and Tables

**Table 1:** Family planning and contraception in women with schizophrenia.

Antipsychotic-Related Concerns at the Pregnancy Planning Stage		
	**Patient concern**	**Recommendations**
**Desire for contraception**	Estrogen levels impact antipsychotic plasma concentrations [24,25,26].	Therapeutic drug monitoring.
**Planning to conceive**	Antipsychotic-induced hyperprolactinemia reduces fertility [17,18,20].	Avoid prolactin-raising antipsychotics.
**Contraceptive efficacy**		
	**Patient concern**	**Recommendations**
**Desire for contraception**	Women with schizophrenia show high rates of unplanned pregnancies [29,30].	Use of long-acting reversible contraceptives or an Intrauterine Device (IUD).
**Pharmacotherapy during pregnancy**		
	**Patient concern**	**Recommendations**
**Planning to conceive**	The perinatal period is an important time to prevent relapse for the health of both the woman and the offspring [33].	Discuss the risks and benefits of treatment options for the mother and her fetus. For many pregnant women, continuation of an antipsychotic medication is imperative for her health and her family.

**Table 2:** Main findings of fertility treatments in women suffering from schizophrenia.

	Patient Concern	Recommendations
**Use of fertility treatments**	Few women with schizophrenia are included in fertility treatments [37,38,39].	To identify and address barriers to accessing fertility treatment for women with schizophrenia.
**Safety of fertility treatments**	There are potential interactions between antipsychotics and medications used in ART [47].	Pharmacodynamic and pharmacokinetic interactions between ART treatments and psychotropic medications should be considered [41].
**Effectiveness of fertility treatments**	Lower treatment success in women with psychotic disorders than in controls [40]. Hyperprolactinemia reduces the effectiveness of fertility treatments [49].	Optimizing healthy lifestyle habits, treating hyperprolactinemia, and providing close follow-up may help to improve fertility rates in people with schizophrenia [49].

**Table 3:** Main findings of studies focusing on maternal health needs of women with schizophrenia.

	Patient Concern	Recommendations
**Prevention of worsening clinical course**	Insomnia, poor hygiene, and suspiciousness are associated with poor mental health [54]	Maintenance of pharmacological and non-pharmacological interventions [53]
**Clinical attention perinatal period**	Psychological interventions can be held to promote trust and improve adherence to follow-up [59]	Development of well-trained multidisciplinary teams [55]
**Support for the mother and children**	Family visits, psychoeducation, and training can improve the health of both mothers and babies [58].	Offering parenting assessment and additional support services to perinatal women with schizophrenia [51,56]
**Anti-stigma interventions**	The stigma of being a mother or caring for children impacts clinical outcomes in schizophrenia [50]	Provide health and legal guidance to mothers to support childcare tasks [50]
**Socioeconomic needs**	Financial stress impacts caring for children [56].	Help with financial resources may help women caring for children [56].

## Data Availability

Not applicable.
